# Spontaneous Swallowing Frequency in Post-Stroke Patients with and Without Oropharyngeal Dysphagia: An Observational Study

**DOI:** 10.1007/s00455-022-10451-3

**Published:** 2022-04-23

**Authors:** Marta Alvarez-Larruy, Noemí Tomsen, Nicolau Guanyabens, Ernest Palomeras, Pere Clavé, Weslania Nascimento

**Affiliations:** 1 Gastrointestinal Physiology Laboratory, Department of Surgery, Hospital de Mataró, Universitat Autónoma de Barcelona, Mataró, Spain; 2 Department of Neurology, Hospital de Mataró, Mataró, Spain; 3 Centro de Investigación Biomédica en Red de Enfermedades Hepáticas Y Digestivas (CIBERehd), Instituto de Salud Carlos III, Barcelona, Spain; 4 Department of Surgery, Hospital de Mataró, Universitat Autònoma de Barcelona, Carretera de Cirera s/n 08304, Mataró, Spain

**Keywords:** Spontaneous swallowing frequency, Stroke, Swallow, Deglutition, Oropharyngeal Dysphagia

## Abstract

Oropharyngeal dysphagia (OD) is a frequent complication after stroke (PSOD) that increases morbidity and mortality. Early detection of PSOD is essential to reduce morbidity and mortality in patients with acute stroke. In recent years, an association between reduced spontaneous swallowing frequency (SSF) and OD has been described. Likewise, the reduction of saliva substance P (SP) concentration has been associated with an increased risk of aspiration and a decrease in SSF. In this study we aimed to compare SSF, salivary SP concentration, hydration and nutritional status in post-stroke (PS) patients with and without OD. We included 45 acute PS patients (4.98 ± 2.80 days from stroke onset, 62.22% men, 71.78 ± 13.46 year). The Volume-Viscosity Swallowing Test (V-VST) was performed for clinical diagnosis of OD. SSF/minute was assessed through 10-min neurophysiological surface recordings including suprahyoid-electromyography and cricothyroid-accelerometry. Saliva samples were collected with a Salivette® to determine SP by ELISA. Hydration status was assessed by bioimpedance. Nutritional status was evaluated by Mini Nutritional Assessment Short Form (MNA-sf) and blood analysis. Twenty-seven PS patients (60%) had OD; 19 (40%), impaired safety of swallow. SSF was significantly reduced in PSOD, 0.23 ± 0.18 and PSOD with impaired safety, 0.22 ± 0.18 vs 0.48 ± 0.29 swallows/minute in PS without OD (PSnOD); (both *p* < 0.005). Nutritional risk was observed in 62.92% PSOD vs 11.11% PSnOD (*p* = 0.007) and visceral protein markers were also significantly reduced in PSOD (*p* < 0.05). Bioimpedance showed intracellular dehydration in 37.50% PSOD vs none in PSnOD. There were no differences for saliva SP concentrations. SSF is significantly reduced in PSOD in comparison with PSnOD. Acute PSOD patients present poor nutritional status, hydropenia, and high risk for respiratory complications.

**Supplementary Information:**

The online version contains supplementary material available at 10.1007/s00455-022-10451-3.

## Introduction

Stroke is the second cause of death in the world [1] and is also a leading cause of disability, which is expected to increase in the coming decades [2] with high socioeconomic costs [3, 4]. Oropharyngeal dysphagia (OD) is one of the most important causes of disability and mortality in patients who have suffered a stroke [1, 2, 5, 6]. According to a study carried out by our group [7], the prevalence of post-stroke (PS) OD on admission was 45%, with up to 38% patients showing clinical signs of impaired safety of swallow. The most prevalent sign of impaired safety of swallow was voice change observed in up to 43.65% [7]. Undetected and untreated patients with OD are at elevated risk of prolonged hospitalization, respiratory infection, pneumonia, malnutrition, dehydration, and death [5, 7–12]. Early identification of post-stroke oropharyngeal dysphagia (PSOD) is fundamental to reduce morbidity and mortality during hospital admission and after hospital discharge [13, 14].

The volume-viscosity swallow test (V-VST), an effort test that uses boluses of different volumes and viscosities to identify clinical signs of impaired efficacy and/or safety of swallow [15], is a validated bedside tool commonly used for clinical diagnosis of PSOD [16]. We recently assessed the psychometrics of V-VST through a systematic review and found that V-VST has good psychometric properties for OD in several phenotypes [16]. Regarding diagnostic accuracy, the V-VST had a sensitivity for OD of 93.17%, 81.39% specificity, and an inter-rater reliability Kappa of 0.77. Also, likelihood ratios (LHR) for OD were 0.08 (LHR–) and 5.01 (LHR +), and the diagnostic odds ratio for OD was 51.18 [16]. Specifically for clinical signs of safety impairment, the V-VST presented a sensitivity of 88.2% and specificity of 64.7%; for pharyngeal residue, it had a sensitivity of 88.4% and specificity at 34.6% [17].

Normal swallowing requires the participation of a widely extended cerebral network with final output motor commands occurring after activation of brainstem circuits involving the nuclei of the central pattern generators [18, 19]. The integrity of this complex top-to-bottom neural axis is necessary for the coordination of the oropharyngeal swallow response during volitional swallows and ideal for reflexive or spontaneous swallows in order to adapt the motor response to the passage of the oral content through the oropharynx [20, 21]. Spontaneous swallowing is, therefore, a protective aerodigestive oligosynaptic brainstem reflex, the primary trigger of which depends on a “peripheral” stimulus (saliva) but could also involve a central source and modulation mechanism [21] although this aspect is still under discussion. Despite evidence that reflexive swallow still occurs in decerebrated animals [18], in humans this reflex response may be modulated by cortical descending influences similar to other brainstem reflexes [19, 22, 23]. Spontaneous swallowing (SS) physiologically contributes to the protection of the respiratory tract by clearing saliva and its impairment could lead to an increase in pharyngeal secretions and the risk of aspiration [24].

Previous studies have shown that spontaneous swallowing frequency (SSF) is reduced in patients with dysphagia secondary to multiple medical conditions (hospitalized older patients, stroke, Parkinson's disease, etc.) [24–28]. In the field of stroke, measurement of SSF has been proposed as a potential index for the screening of dysphagia in acute stroke patients [26]. In the study of Crary et al., for patients with an acute stroke and OD, SSF was also reduced to half (0.23 ± 0.15 swallows/min) that of the subgroup without OD (0.56 ± 0.31 swallows/min); and SSF correlated negatively and significantly with stroke and swallow severity indices but not with age, time from stroke onset, or consciousness level [26]. They found that a SSF lower than 0.40 could identify clinically significant OD with a sensitivity of 96% and a specificity of 68%, and that a 5–10 min sampling window was sufficient to calculate SSF. The authors concluded that SSF has high potential to identify PSOD with psychometric properties equal or superior to clinical screening protocols [29].

Carnaby et al. [30] obtained similar results to Crary et al. [26], showing that patients with acute stroke and dysphagia had a reduction in SSF (0.27 swallows/min) compared to patients with acute stroke and without dysphagia (0.51 swallows/min) at the time of stroke admission. In addition, they showed a reduction in SSF at the time of discharge (0.34 vs 0.47 swallows/min) and an association between reduced SSF and greater severity of dysphagia and stroke, evaluated in terms of disability and institutionalization.

Recently, our group demonstrated a decline in SSF with aging and an increase following peripheral oropharyngeal sensory stimulation [31]. The mean of SSF in young healthy volunteers was around one swallow per minute and was significantly reduced to half by older age but not by gender with a significant negative correlation between age and SSF [31]. Little is known yet about reflexive swallow mechanisms and the role of spontaneous swallowing frequency in OD. As Bulmer et al. [32] found, there is a negative association of SSF with age, which could be implicated in dysphagia. However, they conclude in their review that although dysphagia has been associated with lower SSF there is still high heterogeneity in SSF data and methodologies used to evaluate it, that it is difficult to assess the cause and effect of SSF on dysphagia and that age and comorbidities are potential confounding factors [32].

Saliva plays a role in swallowing, helping digestion, and bolus formation. Saliva quantity and quality can be affected by multiple diseases and medical treatments [33]. In normal conditions daily saliva production ranges between 0.5 and 1.5 L. When unstimulated, saliva flow rate is 0.3–0.4 mL min. Salivary flow is affected by conscious state, decreasing to 0.1 mL/min when sleeping. In addition, an increase is observed while eating (4.0–5.0 mL/min) [33]. Substance P (SP), is a neuropeptide commonly present in saliva that acts as a neurotransmitter in the pharyngeal mucosa in response to local stimuli and is thought to have an important role in the swallowing reflex, following the mechanism of action described for the cough reflex [34]. Previous studies reported that SP concentration in sputum is reduced in older and PS patients with aspiration pneumonia [35, 36] and that a low concentration of SP in saliva would discriminate against those patients with reduced SSF and pharyngeal sensitivity [25, 37]. SP has also been used as a potential marker of neurorehabilitation peripheral treatments for OD [38, 39].

Because the neural control and modulation of spontaneous swallowing and the mechanisms involved are still largely unknown in humans, in the present study we aimed to compare SSF, salivary SP concentration, hydration and nutritional status of PS patients with and without oropharyngeal dysphagia. We hypothesized that PSOD patients will present reduced SSF and SP levels, and poor nutritional and hydration status when compared to PS patients without OD (PSnOD).

## Materials and Methods

### Study Population

Forty-five PS patients were recruited during their admission to the Neurology Department at the spital de Mataró, Barcelona, Spain. The inclusion criteria were to be 18 years old or older, to have hemorrhagic or ischemic unilateral stroke diagnosed by a neurologist using clinical and imaging tests—computed tomography or magnetic resonance imaging—no more than 15 days from stroke onset. Exclusion criteria were to have a life expectancy of less than 3 months, severe dementia (Global Deterioration Scale [40] greater than 5) or suspected or confirmed SARS-COV-2 infection (by RT-PCR 48 h before the visit). All included patients tested negative for Sars-CoV-2 on admission.

### Study Design

**Fig. 1:**
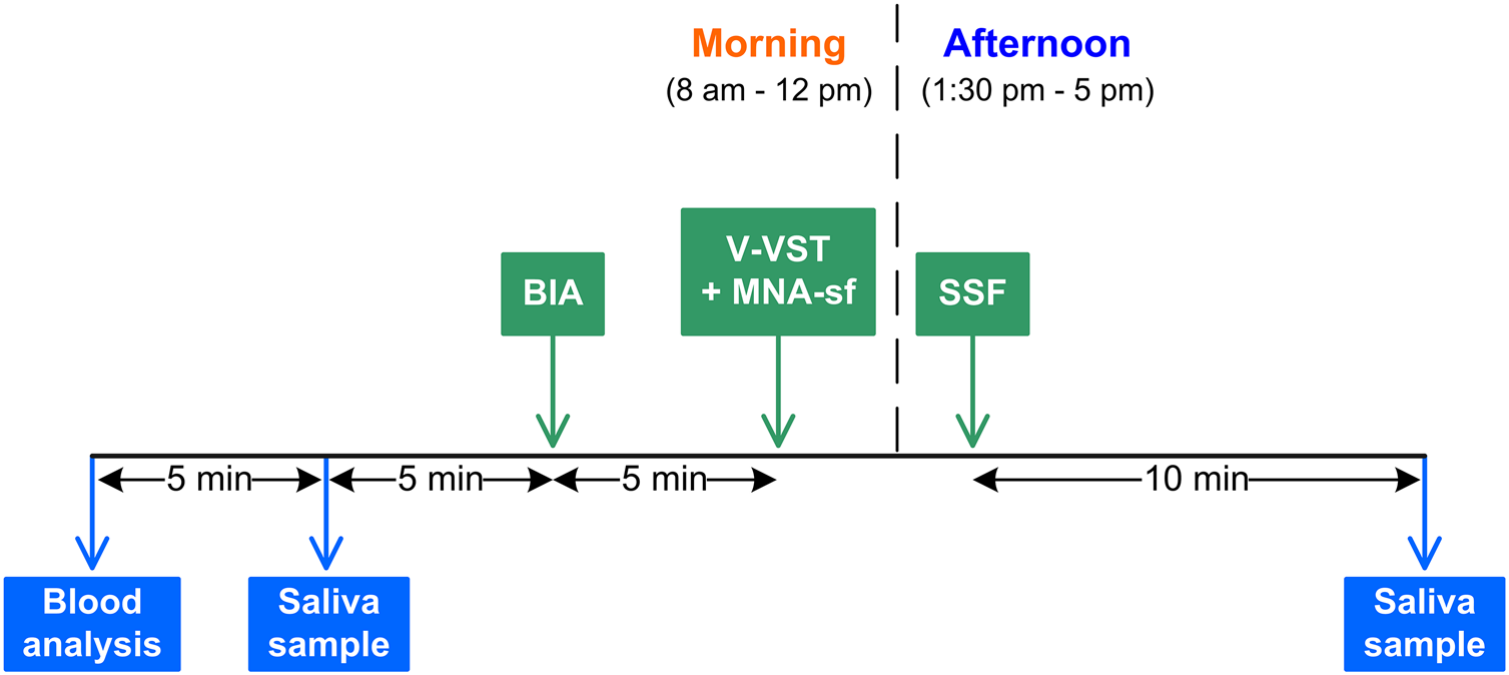
Study design. *BIA* bioimpedance, *V-VST* volume viscosity swallow test, *MNA-SF* mini nutritional assessment-short form

The study was an observational transversal study conducted with the cooperation of researchers from the Gastrointestinal Physiology Lab and the Neurology Department of Hospital de Mataró. The study protocol was approved by the Ethical Committee of the Hospital de Mataró (CEIm 74/20) and performed according to the rules of the Declaration of Helsinki. All the participants signed the informed consent. Patients who agreed to participate had two visits performed one in the morning and the other in the afternoon of the same day (Fig. 1). During the morning visit a sample of blood and saliva were collected, the nutritional status was screened with the Mini Nutritional Assessment short form (MNA-sf) [41], and body composition was assessed by bioimpedance. In addition, all PS patients were screened using V-VST in order to assess the clinical signs of impaired safety and efficacy of swallow and to classify them into having dysphagia (PSOD) or not having dysphagia (PSnOD). In the afternoon, the SSF was recorded for 10 min and an additional saliva sample was collected. In addition, demographic characteristics, medical history, usual medication, functional status, and stroke severity scales were also collected on admission. Baseline functional status was defined according to the modified Rankin scale (mRS) [42] and the Barthel index [43]. Stroke characteristics included the time between stroke onset and assistance, National institute of Health Stroke Scale (NIHSS) [44], current and previous neuroimaging lesions, Oxford stroke classification [45], lateralization, affected arterial territory, and ischemic or hemorrhagic stroke etiology.

### Study Variables and Procedures

#### Volume-Viscosity Swallowing Test (V-VST)

All patients were assessed with the V-VST [17]. The swallowing function was assessed while swallowing boluses of 5, 10, or 20 mL of < 50 mPa·s (liquid), 250 mPa·s (nectar), or 800 mPa·s (honey). 250 and 800 mPa·s were obtained by adding 2.0 g and 5.5 g of Nutilis Clear (Nutricia, Barcelona, Spain), respectively, to 100 mL of water. In order to analyze the effect of oropharyngeal dysphagia on the variables, the patients were divided in two main groups after the V-VST: post-stroke patients with dysphagia in one group (PSOD) and without dysphagia in the other (PSnOD). Patients who presented any sign of safety and/or efficacy impairments, except for piecemeal deglutition with 20 ml at 800 mPa.s (honey viscosity), were included in the PSOD group. A subgroup was also considered with patients with clinical signs of impaired safety of swallow according the V-VST.

#### Neurophysiological Assessment of Spontaneous Swallow (SS)

The neurophysiological recording method for volitional swallow assessment previously described by Ertekin et al. [46, 47] was used to characterize SS according to kinematic measures. Surface electromyography (EMG) and accelerometry were used to record suprahyoid complex and laryngeal activation, respectively, as previously described by our team [31]. A pair of 2 × 2 cm adhesive EMG electrodes were positioned over the suprahyoid muscles, while a 1.5 × 1.5 cm omnidirectional accelerometer (Pro-Tech Limb Movement Sensors-PLM, RespironicsⓇ Inc., Nevada, USA) was placed over the cricothyroid cartilage. Online recordings from both the EMG signals (bandpass 100–5000 Hz) and accelerometer activity (bandpass < 0.001–10 Hz) were entered into a polygraphic recording system (Biopac Systems Inc., California, USA). An additional online calculation channel displaying the rectified and integrated activity from the EMG channel was built. All recordings were analyzed offline using the AcqKnowledge software (Biopac, USA), which displayed a visual trace of all three channels. Spontaneous swallows were considered to be present when the following two criteria were fulfilled: (1) the activity recorded on the EMG and the accelerometer’s channels was compatible with a typical neurophysiological swallow configuration as described [46], and (2) the previous recording activity was consistent with SS through visual inspection by the investigator. A baseline 10 min recording was performed in each patient for SS characterization.

##### Neurophysiological Data Reduction and Analysis

After the 10’-recording, we obtained the following data for each individual: total number of SS and number of swallows per minute. Our primary physiological outcome was the SSF, which was calculated as the total number of SS during the recording divided by 10 (expressed as swallows/min). Additionally, in order to characterize SS performance of study populations, the following kinematic measures were calculated in patients for each SS [31]: amplitude (distance from highest to lowest peak of the EMG signal, expressed in volts), duration, delta T (∆T, time from the start of the EMG signal to the end in seconds), and area under the curve (AUC, amplitude and ∆T integral, in volts·seconds).

#### Saliva Sample Collection and SP Quantification

Saliva samples were collected in the morning (from 8 am to 12 noon) and also in the afternoon (from 1:30 to 5 pm) from all participants. A Salivette® (Sarstedt, Nuembrecht, Germany) was used to collect the saliva, putting the swab under the tongue of the patient for 5 min. After the centrifugation (at 2600 rpm for 2 min) the clean sample was stored at −80 °C until the determination of SP using specific commercial enzyme-linked immunosorbent assay (ELISA) kits: Substance P Parameter Assay Kit (ref. KGE007; R&D systems, Minneapolis, MN, USA).

#### Hydration Status. Body Composition

Body composition was measured using bioimpedance [37, 48] with the InBody S10 (InBody Co., Ltd., Seoul, Korea), by placing an electrode on the thumb and ring finger of each hand and one more pair on each ankle with the patient in a lying or sitting position depending on their condition. The parameters collected were the percentage of total body water, extracellular water, intracellular water, fat mass and muscle mass and the phase angle (in degrees). The reference ranges for total body water, extracellular water, intracellular water and fat and muscle mass were automatically calculated by the equipment according to the age, sex, height, and weight of each participant. Phase angle normality level was established as equal or greater than 4.40º according to our previous studies [37]. Reduction of the phase angle is related to cell dehydration that compromises the integrity of the plasmatic membrane, altering its capacity to separate charges of the bioimpedance electric current [37]. Phase angle is also linked to the quality of the muscle function in older adults [49, 50].

#### Nutritional Status. Mini Nutritional Assessment Short Form (MNA-SF) and Blood Analysis

In order to screen for risk of malnourishment, the patients completed the MNA-sf [41]. If a patient had difficulty with comprehension/expression, a family member or caregiver was interviewed. In addition, blood samples were collected as part of routine care during the patient admission. From the blood analysis, we collected data on the values of albumin, prealbumin, protein, lymphocytes, and total cholesterol. Blood analytical parameter reference intervals were taken from the Reference Laboratory of Catalonia [51]: for albumin they were 3.5 to 5.2 g/dL; for prealbumin, 20.0 to 40.0 g/dL; total protein level was considered normal between 6.0 and 8.3 g/dL; lymphocytes, between 1 × 10^3^ and 3 × 10^3^/µL; and total cholesterol, 120.0 to 200.0 mg/dL.

#### Functional Outcome Scale Score (FOIS) [52]

A seven-point ordinal scale was used to document the functional level of oral intake of food and liquid based on the diet prescriptions from the assessment day.

### Power Analysis

We conducted a sample size analysis before starting data collection: Accepting an alpha risk of 0.05 and a beta risk of 0.2 in a two-sided test, a minimum of 20 persons are necessary in the first group and 20 in the second to find a statistically significant difference in proportion, expected to be 0.35 in the OD group and 0.8 in the control group. A drop-out rate of 15% was anticipated. This was calculated on the expected difference in SSF between patients with and without OD.

### Data Analysis and Statistical Methods

Continuous data were expressed as mean ± standard deviation (SD) and categorical data as absolute or relative frequencies. Continuous data were analyzed by unpaired t-test (PSOD vs PSnOD, PSOD with impaired safety vs PSnOD) or pair t-test (pre-admission vs discharge) while categorical data were compared by Fisher’s exact test. A non-parametric test was performed for non-Gaussian data. Receiver-operator characteristic (ROC) curves were drawn to explore the cut-off SSF to assess the accuracy of the clinical diagnosis of dysphagia and impaired safety of swallow. Statistically significant differences were considered when p-value was less than 0.05 and all the analyses were performed using GraphPad Prism 6 (GraphPad Software, San Diego, CA, USA).

## Results

### Demographic and Stroke Characteristics

Forty-five post-stroke patients, 28 men (62.22%), with a mean age of 71.78 (13.46) years old were included in this study. Mean time between the stroke onset and inclusion in the study was 4.98 (2.80) days. Severity of strokes according to NIHSS was mild (Table 1) (Table SI).

**Table 1:** Demographic and neuroimaging characteristics of the study population

	All	PSOD	PSnOD	*p*-value
N	45	27	18	
Sociodemographic Data				
Sex (% men)	62.22 (28)	55.56 (15)	72.22 (13)	0.3513
Age (years)	71.78 ± 13.46	76.81 ± 12.34	64.22 ± 11.37	0.0015
Barthel Index pre-admission (mean ± SD)	95.56 ± 10.71	93.33 ± 13.08	98.89 ± 4.71	0.0818
Barthel Index admission (mean ± SD)	60.56 ± 32.01	47.48 ± 31.97	79.72 ± 22.39	0.0003
MNA-sf (mean ± SD)	10.82 ± 1.74	10.22 ± 1.93	11.72 ± 0.80	0.0009
NIHSS (mean ± SD)	5.09 ± 5.62	7.52 ± 6.17	1.44 ± 1.34	< 0.0001
Stroke characteristics				
Days from ictus to evaluation (mean ± SD)	4.98 ± 2.80	4.39 ± 2.48	5.37 ± 3.03	0.2326
mRS pre-admission (%)				
0–2	80.00	70.37	94.44	0.0644
3–5	20.00	29.63	5.56	0.0644
Stroke subtype (%)				
Ischemic	84.44	81.48	88.89	0.6844
Hemorrhagic	15.56	18.52	11.11	0.6844
Acute lesions in neuroimaging (%)				
No lesions	4.44	0	11.11	0.1545
Territorial infarction	68.89	77.78	72.22	1.000
Lacunar infarction	11.11	11.11	11.11	1.000
Intraparenchymal hemorrhage	15.56	18.52	11.11	0.6844
Lateralization (%)				
Right hemisphere	42.22	44.44	38.89	0.7660
Left hemisphere	44.44	44.44	44.44	1.000
Brain stem	13.33	11.11	16.67	0.6703
Oxford (%)				
PACI	24.44	18.52	33.33	0.3040
TACI	24.44	37.04	5.56	0.0307
POCI	6.67	3.70	16.67	0.2862
LACI	42.22	40.74	44.44	1.000

*PACI* partial anterior circulation infarcts, *TACI* total anterior circulation infarcts, *POCI* posterior circulation infarcts, *LACI* lacunar infarcts

Most patients had ischemic (84.44%) strokes and also most of them were supratentorial (86.66%) strokes affecting purely the anterior circulation brain territory (81.57%). Proportion of left and right hemisphere involvement was the same. The majority of patients (46.67%) also showed leukoaraiosis. The etiology of ischemic stroke was atherothrombotic in 26.32% of patients, cardioembolic in 23.68%, lacunar in 15.79%, and indeterminate in 34.21%. All hemorrhagic strokes (*n* = 7; 15.56%) were of hypertensive etiology.

### PSOD versus PSnOD Demographic and Clinical Characteristics

PSOD patients were significantly older than PSnOD patients (*p* = 0.0015), (Table 1). However, there were no differences in sex distribution between groups. The post-stroke level of independence for daily activities (Barthel Index) decreased in both groups with significant differences (*p* = 0.0003). In addition, the severity of stroke was significantly higher in PSOD, demonstrated by moderate stroke severity against minor stroke in the PSnOD group (*p* =  < 0.0001).

### Volume-Viscosity Swallowing Test (V-VST)

#### Post-Stroke Patients Without Dysphagia (PSnOD)

Eighteen patients did not present impaired efficacy or safety of swallowing during the V-VST.

#### Post-Stroke Patients with Dysphagia (PSOD)

Twenty-seven patients (60%) presented clinical signs of OD according to the V-VST. Impaired efficacy and impaired safety was observed in 42.20%. Eight (29.63%) patients presented only efficacy impairment and 19 (70.37%) had safety impairment. From the group with safety impairment, 6 (31.58%) presented only safety impairment and 13 (68.42%) both safety and efficacy impairment.

**Fig. 2:**
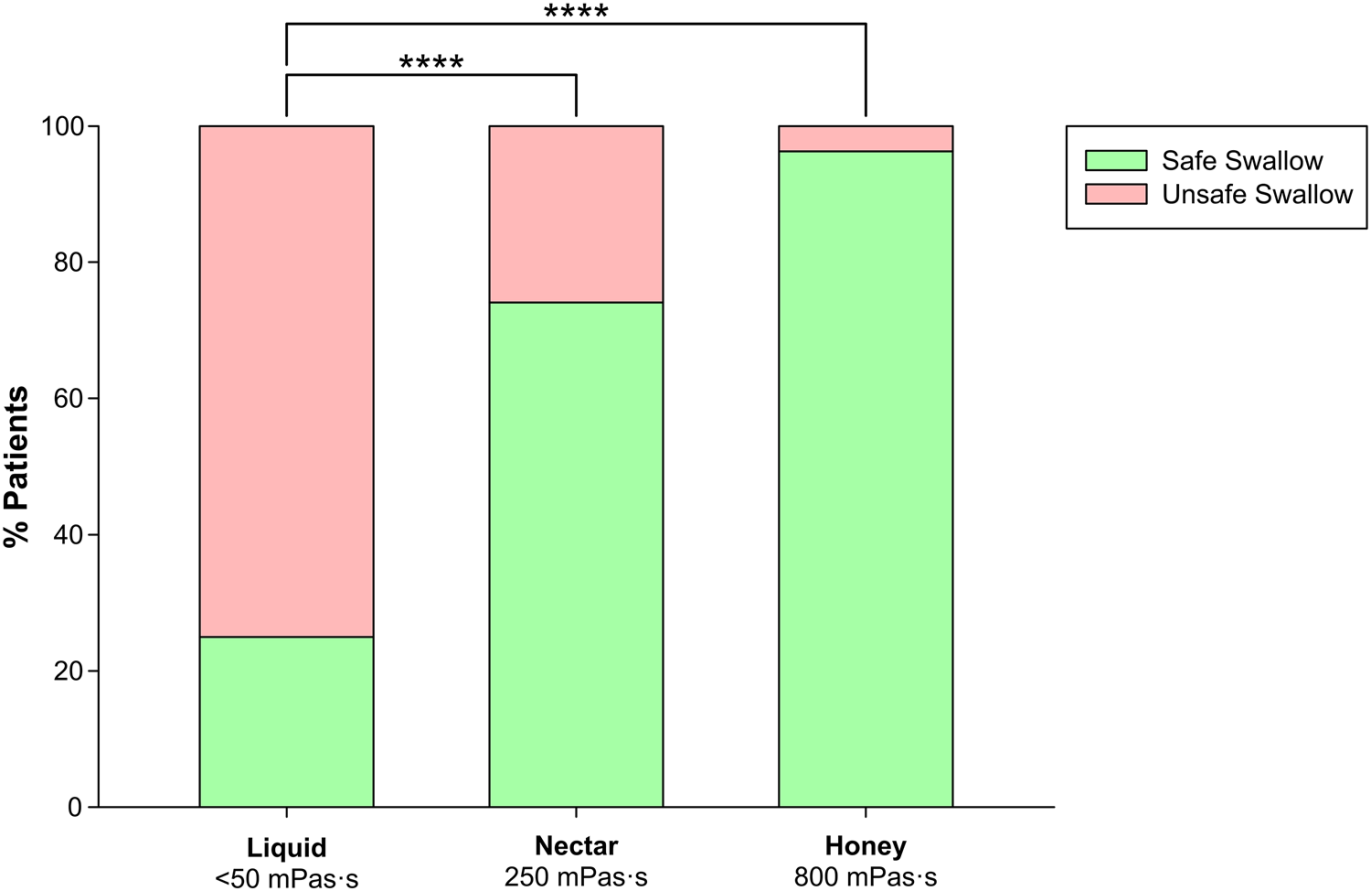
Effect of fluid thickening at increased shear viscosity values on safety (250 mPa.s vs thin liquid *p* < 0.0001; 800 mPa.s vs thin liquid *p* < 0.0001) of swallow in PSOD. Note the strong therapeutic effect of increasing bolus viscosity on safety of swallow

Regarding impaired safety, voice changes were present in 8.89% with nectar and 7.89% with liquid (*p* = 0.1371). Oxygen desaturation was observed in 2.22% with nectar and honey (*p* = 0.6512). Cough was seen in 6.67% for nectar viscosity and 42.11% with liquid (nectar vs liquid *p* < 0.0001; honey vs liquid *p* < 0.0001), demonstrating a therapeutic effect of this thickening agent improving swallow safety (Fig. 2).

For impaired efficacy, lip seal impairment was observed in 6.67% with nectar and 4.44% with honey viscosity (*p* = 0.2875). Oral residue reached 6.67% with nectar and 11.11% with honey (*p* = 0.1130). Piecemeal deglutition was seen in 37.78% with nectar and honey, and 10.53% with liquid (nectar vs liquid *p* = 0.0053; honey vs liquid *p* = 0.0053; nectar vs honey *p* = 1.000). Pharyngeal residue was detected in 2.22% with nectar and 11.11% with honey (*p* = 0.0362; nectar vs liquid *p* = 1.000; honey vs liquid *p* = 0.2599; nectar vs honey *p* = 0.2028).

### Spontaneous Swallow Characterization in PSnOD and PSOD Patients. SSF in Patients with PSOD and Impaired Safety of Swallow

SSF was 0.48 ± 0.29 swallows per minute in PSnOD patients, and was significantly reduced to half (0.23 ± 0.18 swallows per minute) in PSOD patients (*N* = 27, *p* = 0.0017). In PSnOD, the mean amplitude was 0.64 ± 1.62 V, the mean delta T 1.66 ± 0.27 s, and the mean AUC 0.07 ± 0.07 V·seconds. For PSOD, the mean amplitude was 0.76 ± 1.25 V, the mean delta T was 1.81 ± 0.90 s, and the mean AUC 0.14 ± 0.22 V-second. Considering PSOD with clinical signs of impairment in swallow safety (*n* = 19), we observed that SSF was 0.22 ± 0.18 swallows per minute, and also significantly decreased by half in comparison with PSnOD (*p* = 0.0024). For PSOD with safety impairment, the mean amplitude was 0.42 ± 0.50 V, the mean delta T was 1.63 ± 0.40 s, and the mean AUC 0.07 ± 0.05 V-second. No significant differences on these metrics were observed when comparing PSOD or PSOD with safety impairments vs PSnOD (*p* > 0.05).

### ROC Curve to Predict Oropharyngeal Dysphagia and Impaired Swallow Safety

**Fig. 3:**
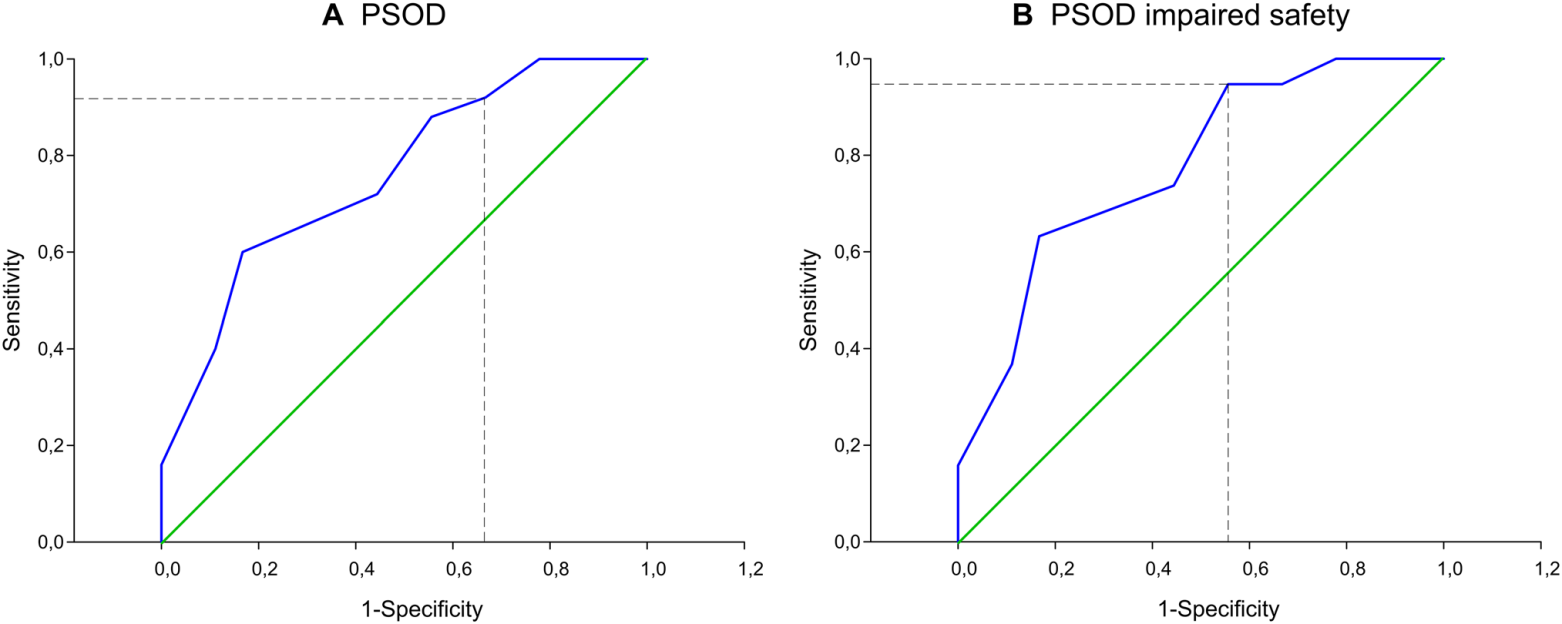
SSF receiver-operator characteristic (ROC) curve showing spontaneous swallowing frequency sensitivity/ specificity for oropharyngeal dysphagia (**A**) or impaired safety of swallow (**B**). **A**. Depicted is the cut-off value of 0.55 swallows per minute for OD. **B**. Depicted is the cut-off value of 0.45 swallows per minute for impaired safety of swallow. *PSOD* post-stroke patients with oropharyngeal dysphagia, *SSF* spontaneous swallowing frequency

ROC curve analysis of SSF was developed to assess the accuracy of the clinical diagnosis of dysphagia and impaired safety of swallow used to predict dysphagia and to select the best cut-off value (Fig. 3). Considering the SSF, the cut-off value to predict dysphagia in PSOD was 0.55 swallows/minute, with a sensitivity of 0.92, a specificity of 0.33, and AUC of 0.76 (95% CI: 0.62–0.90). However, when considering PSOD with safety impairment, the cut-off value to predict unsafe swallows was 0.45 swallows/minute, with a sensitivity of 0.95, a specificity of 0.44, and AUC of 0.76 (95% CI: 0.63–0.90).

### SP Levels in Saliva

In the saliva collected in the morning, SP concentration was 62.02 ± 26.23 pg/ml in the PSnOD group and 60.55 ± 26.08 pg/ml for PSOD. In the afternoon, the concentration for PsnOD patients was 76.28 ± 57.88 pg/ml and 78.80 ± 63.19 pg/ml in the PSOD group. No differences were found between groups for both morning (*p* = 0.7524) and afternoon (*p* = 0.4121) SP concentration in saliva.

### Nutritional Status. MNA-SF and Blood Biomarkers

The mean MNA-sf score was significantly reduced in PSOD (10.22 ± 1.97) when compared to the PSnOD group (11.72 ± 0.83) (*p* = 0.0009). Normal nutritional status was observed in 37.04% of PSOD vs 88.89% of PSnOD (*p* = 0.007). Also, high prevalence of risk of malnourishment was observed in the PSOD group (PSOD 48.15% vs PSnOD 11.11%; *p* = 0.0118). There were no differences regarding the prevalence of malnutrition for both groups (PSOD 14.81% vs PSnOD 0%; *p* = 0.1383). Blood biomarkers showed lower mean albumin (*p* = 0.0224), prealbumin (*p* = 0.0128), and protein (*p* = 0.0419) in PSOD (Table 2). There were no differences for lymphocytes (*p* = 0.3142) and total cholesterol (*p* = 0.1483) levels. These results show hypermetabolism and changes in nutritional status. Also, total protein, albumin, and prealbumin are good markers for acute changes in nutritional status.

**Table 2:** Serum nutritional biomarkers in PSOD versus PSnOD

	PSOD	PSnOD	*p*-value
Albumin (g/dL)	3.75 ± 0.53	4.28 ± 0.93	0.0224
Prealbumin (g/dL)	17.99 ± 5.87	22.71 ± 5.10	0.0128
Total Proteins (g/dL)	6.61 ± 0.46	6.88 ± 0.37	0.0419
Lymphocytes (µL)	1.97 ± 1.07	2.18 ± 0.84	0.3142
Total cholesterol (mg/dL)	147.29 ± 38.81	162.14 ± 38.52	0.1483

*PSOD* post-stroke patients with oropharyngeal dysphagia, *PSnOD* post-stroke patients without oropharyngeal dysphagia

### Hydration Status. Bioimpedance

Bioimpedance measurements showed that there was no alteration in the mean angle phase in PSnOD group; however, 37.50% of PSOD presented impairment suggesting intracellular dehydration (*p* = 0.0054). (Table 3). Regarding bioimpedance nutritional measurements/body composition, we observed a tendency, not statistically significant, to lower percentage levels of total water, intracellular water and muscle mass in the PSOD group. There was no difference for extracellular water and fat mass between groups.

**Table 3:** Body composition in PSOD versus PSnOD

	PSOD	PSnOD	*p*-value
Total water (%)	35.13 ± 9.49	39.00 ± 7.01	0.0664
Intracellular water (%)	21.47 ± 5.87	24.23 ± 4.57	0.0570
Extracellular water (%)	13.66 ± 3.64	14.77 ± 2.48	0.0997
Muscle mass (%)	26.00 ± 7.65	29.59 ± 5.95	0.0552
Fat mass (%)	30.03 ± 14.04	27.91 ± 12.87	0.7595
Phase angle (degrees)	4.96 ± 1.03	6.01 ± 1.15	0.0054

*PSOD p*ost-stroke patients with oropharyngeal dysphagia, *PSnOD* post-stroke patients without oropharyngeal dysphagia

### FOIS

All patients included in the study were fed exclusively by mouth but there was a significant difference between swallowing functionality between groups (< 0.0001) showing that PSOD patients were in a need for modified texture diet and fluid thickening, and this was not an issue for the PSnOD group. All PSnOD patients were eating a normal texture diet with thin liquid for hydration [53] except for one, who needed an level E diet (easy to chew) because of tooth loss; they presented a mean FOIS score of 6.89 ± 0.46. The mean FOIS score for the PSOD group however was 4.70 ± 0.81 (*p* < 0.005 vs PSnOD), showing that the majority of them needed a texture modified diet level E (fork mashable) or C (puree like) (*N* = 18); 10 patients (55.56%) needed nectar (250 mPa·s); 8 patients (44.44%) needed honey (800 mPa·s) viscosity to maintain a safe swallow while swallowing fluids and 9 patients did not need thickened fluids.

## Discussion

The main objective of this study was to assess whether SSF is decreased in patients with an acute stroke and dysphagia. The secondary objectives were to analyze whether there is a decrease in the levels of substance P in saliva and a worse nutritional and hydration status in patients with an acute stroke and dysphagia. The prevalence of OD among acute stroke patients was 60.00%, higher than in our previous studies [7]. This might be attributed to 1) the higher severity of stroke events in this study than in the previous one (NIHSS mean of 5 compared to 2) possibly due to the fact that patients with mild stroke were not admitted to neurology ward during COVID-19 pandemia, and 3) that all patients in this study were evaluated by the same experienced professional (WN). Regarding the type, topology, and etiology of the stroke, we obtained very similar characteristics to those described in our previous studies and in the literature [7]. Both impaired efficacy and impaired safety of swallow were observed in 42.20% of the PSOD patients in the current study. The main sign of impaired safety was cough and the main sign of impaired efficacy was piecemeal deglutition.

The main result of this study is that SSF was markedly reduced in PSOD patients compared to PSnOD, (0.23 ± 0.18 swallows/minute) compared to PSnOD (0.48 ± 0.29 swallows/minute). These results are very similar to those obtained by Crary et al. [26], who also found a reduction to half of SSF (0.23 ± 0.15 swallows/min in PSOD patients vs 0.56 ± 0.31 swallows/min in PSnOD patients), and those obtained by Carnaby et al. [30] (0.27 ± 0.22 swallows/min vs 0.51 ± 0.30 swallows/min). In addition, Crary et al. [29] suggested that SSF may be a useful screening tool, but it lacked the specificity for a definitive diagnosis. Similarly to Crary et al.’s [26] study, we made a ROC curve to assess the accuracy of measuring SSF as a screening tool for dysphagia and impaired safety of swallow, using the cut-off values of 0.55 swallows/minute for PSOD and 0.45 swallows/minute for PSOD with impaired safety. The results showed that SSF has quite good diagnostic accuracy, and a reduction in SSF presents high sensitivity, but low specificity in PSOD.

In previous studies we also found a significant decrease in pharyngeal sensory function associated with aging, much more severe in older patients with dysphagia [54]. In patients with PSOD and impaired safety of swallow, videofluoroscopic studies identified delayed laryngeal vestibule closure time as the critical event causing penetrations and aspirations, and our neurophysiological evaluation demonstrated prevalent impairments with disrupted integration of both pharyngeal sensory inputs and reduced cortical excitability of efferent pathways [55].

The reduction in SSF we thought could be due to the decrease in the pharyngeal sensory stimulus to the central pattern generator secondary to the stroke. Reduced SSF could lead to an increase in the accumulation of oropharyngeal secretions and, therefore, to an increased risk of aspiration and pneumonia [24]. We also found in previous studies that increasing pharyngeal sensory input in post-stroke dysphagic patients by using TRPV1 (transient receptor potential cation channel subfamily V member 1) stimulants almost doubled SSF, further supporting the role of pharyngeal sensory stimulation in the modulation of SSF and the potential use of SSF as a relevant metric in assessing the pharmacological stimulation of swallow response in PSOD [31].

On the other hand there wasn’t a significant difference for SP levels in saliva in PSOD vs PSnOD. Nutritional status was poor in up to 60.00% of PSOD patients and dehydration was observed in 37.50% of them. These results further show PSOD patients are at great risk of malnutrition, dehydration, and respiratory complications.

One of the open questions about the study of the SSF is what is the most appropriate method to evaluate it [32]. Our study not only demonstrates the reproducibility of the previous studies [26, 29, 30], but also the reproducibility by means of another SSF evaluation technique. While previous studies [26, 29, 30] measured SSF with an acoustic recording obtained through a miniature microphone, in our study SSF was measured with EMG. More studies are required to analyze whether SSF could be a screening indicator for dysphagia in acute stroke in combination with more specific clinical and/or instrumental assessments.

Our study presents some limitations. We included a moderate sample size of 45 post-stroke patients which possibly limited the interpretation of the statistical results. A larger sample size is necessary to study this topic in greater depth. In addition, carrying out the study during COVID-19 pandemia could have generated a bias toward the selection of patients with more severe strokes than in our previous studies. Furthermore, in this study dysphagia was determined by V-VST which, despite being a test with excellent psychometric properties for clinical diagnosis of OD, is not the gold standard test for OD, which is the videofluoroscopy. In addition, although the V-VST uses some clinical symptoms, it also uses several signs to identify impaired safety and efficacy of swallow. Finally, although the role of SP in swallowing is still unknown, there are some studies describing its relationship with several complications associated with OD, such as aspiration pneumonia, decreased SSF, and impaired pharyngeal sensitivity [25], [35–37]. Finally, it is important to note that there was only a small number of patients who had enough saliva to be analyzed using ELISA affecting, as a consequence, the statistical power of the salivary neuropeptides results.

For the future, the psychometrics of SSF as a screening method for PSOD show very good sensitivity and limited specificity that suggests the need for its combination with more specific clinical and/or instrumental methods for PSOD. In addition, and taken together with our previous studies on SSF and its potential relation with impaired pharyngeal sensory function, we believe SSF might become a method to assess the effect of pharyngeal pharmacologic stimulation on the swallow response in PSOD.

## Summary

In summary, SSF is significantly reduced in PSOD, and has a high prevalence of clinical signs of impaired safety of swallow. Our study further confirms that acute PSOD patients present hydropenia, poor nutritional status, and high risk for respiratory complications.

## Supplementary Information

Below is the link to the electronic supplementary material.

Supplementary file1 (PDF 422 KB)
