# Supplementary material for: Spontaneous Swallowing Frequency in Post-Stroke Patients with and Without Oropharyngeal Dysphagia: An Observational Study
Source: Dysphagia. 2022 Apr 23;38(1):200–10. doi: 10.1007/s00455-022-10451-3 (PMC9034075; doi:10.1007/s00455-022-10451-3)
Supplement: Supplementary file 1 — Supplementary file1 (PDF 422 KB) [file 455_2022_10451_MOESM1_ESM.pdf]

Supplementary material

Table I: Stroke characteristics.

	All	PSOD	PSnOD	p-value
**N**	**45**	**27**	**18**	
**Chronic lesions in neuroimaging (%)**				
No chronic lesions	35.56	33.33	38.88	0.7578
Territorial cerebral infarction	8.89	11.11	5.55	0.6396
Lacunar infarction	28.89	29.62	27.78	1.000
Leucoaraiosis	46.67	51.85	38.89	0.5434
**Vascular territory - of ischemic strokes (%)**				
ACM	81.58	81.82	81.25	1.000
ACP	2.63	4.55	0.0	1.000
AChA	2.63	0.0	6.25	0.4359
Vertebrobasilar	10.52	13.64	6.25	0.6133
Indeterminate	2.63	0.0	6.25	0.4500
**Infarct etiology (%)**				
Atherothrombotic	26.32	27.27	25.00	1.000
Cardioembolic	23.68	27.27	18.75	0.7060
Lacunar	15.79	18.19	18.75	1.000
Indeterminate	34.21	27.27	37.50	0.7249
**Hemorrhage etiology (%)**				
Hypertension	100.00	100.00	100.00	1.000

PSOD - Post-stroke patients with oropharyngeal dysphagia; PSnOD, Post-stroke patients without oropharyngeal dysphagia
